# Prevention of postoperative intrapericardial adhesion by dextrin hydrogel

**DOI:** 10.1007/s11748-020-01581-2

**Published:** 2021-01-08

**Authors:** Satoshi Kikusaki, Kazuyoshi Takagi, Takahiro Shojima, Kosuke Saku, Tomofumi Fukuda, Atsunobu Oryoji, Tomoyuki Anegawa, Yoshihiro Fukumoto, Hiroki Aoki, Hiroyuki Tanaka

**Affiliations:** 1 Division of Cardiovascular Surgery, Department of Surgery, Kurume University School of Medicine, Kurume, Fukuoka 830-0011 Japan; 2 Division of Cardiovascular Medicine, Department of Internal Medicine, Kurume University School of Medicine, Kurume, Fukuoka 830-0011 Japan; 3 Cardiovascular Research Institute, Kurume University, 67 Asahimachi, Kurume, Fukuoka 830-0011 Japan

**Keywords:** Intrapericardial adhesion, Dextrin hydrogel, Inflammation, Animal model

## Abstract

**Objective:**

Postoperative intrapericardial adhesion increases the risk of complications in patients undergoing reoperation. We investigated the effect of a bioabsorbable dextrin hydrogel (DHG) on the formation of intrapericardial adhesions.

**Methods:**

Intrapericardial adhesion was surgically induced in Japanese white rabbits with DHG treatment (Adh + DHG) or without DHG treatment (Adh). The sham group was not treated with DHG and intrapericardial adhesion was not induced. The extent of intrapericardial adhesion was assessed by adhesion scoring and crystal violet staining of the pericardial cavity. Bromodeoxyuridine (BrdU) uptake assay was performed to assess the proliferative response to the injury in the tissue beneath the intrapericardial adhesion.

**Results:**

The Adh + DHG group showed looser intrapericardial adhesions compared to the Adh group. The adhesion area of the Adh + DHG group was 4.6 ± 2.2%, whereas that of the Adh group was 32.6 ± 6.4% at the end of the 28-day observation period (*p* < 0.01). The induction of intrapericardial adhesion resulted in a proliferative response mainly in the cardiac tissue just beneath the adhesion. There were 48.6 ± 10.7 cells/0.1 mm^2^ BrdU-positive cells in the Adh + DHG group and 135.7 ± 23.8 cells/0.1 mm^2^ BrdU-positive cells in the Adh group on day 28 (*p* < 0.05).

**Conclusion:**

These findings indicate that DHG effectively prevented intrapericardial adhesion in this model.

## Introduction

Cardiovascular diseases are becoming increasingly prevalent in our aging society. Since the risk factors, including age, are common to various cardiovascular diseases, the chances of the same patient presenting multiple cardiovascular diseases is high [1]. It is estimated that 10–20% of the patients who undergo cardiac surgery, such as aortic valve replacement or coronary artery bypass grafting, need reoperation [2], and the proportion of patients needing reoperative cardiovascular surgery is increasing [3]. In addition, patients with congenital heart diseases frequently undergo multiple stages of surgical repair. Postoperative intrapericardial adhesion is a common phenomenon in cardiovascular surgery, which increases the risk of complications including organ injury, bleeding, prolonged cardiopulmonary bypass time, and increased surgical mortality [4–7]. Therefore, the problem of postoperative intrapericardial adhesion needs to be addressed to improve the outcome of patients who might undergo repeated surgery.

The sequence of events in postoperative intrapericardial adhesion has been well characterized [8]. The initial event is the detachment of pericardial mesothelial cells (PMCs) and exposure of the underlying tissue. Concomitantly, vascular congestion, tissue edema, and migration of inflammatory cells occur. Due to the bleeding and the loss of PMCs, fibrin accumulates at the denuded areas, followed by the deposition of collagen fibers resulting in the formation of firm adhesive connective tissue. During this process, PMCs and fibroblasts proliferate to promote healing and fibrosis of the injured tissue [8, 9].

Other than surgical technique to minimize the injury and removal of the blood and clots, several approaches have been proposed to prevent intrapericardial adhesion, including pharmacological suppression of inflammation and cell proliferation, and placement of physical barriers [8]. However, pharmacological interventions are still at an experimental stage and may not be necessarily beneficial as inflammatory responses and cell proliferation are essential for wound healing after surgery [9, 10]. Placing a physical barrier seems a feasible option to prevent postoperative tissue adhesion. Indeed, bioabsorbable dextrin hydrogel (DHG) has been approved in Japan for the prevention of intraperitoneal adhesions [11–15]. A layer of DHG on the surface of the tissue acts as a physical barrier against blood, clots, fibrin, or other tissues, thereby preventing intraperitoneal adhesion [14]. On the other hand, non-absorbable artificial materials seem ineffective in preventing or at times even worsen the inflammatory response and tissue adhesion [16, 17]. The effect of DHG in preventing intrapericardial adhesion has not been reported yet. We thus investigated its effect in an animal model of intrapericardial adhesion.

## Materials and methods

### Animals and surgical procedures

The experimental protocol was approved by the Animal Experiment Committee of Kurume University School of Medicine, and animal care and usage were performed in accordance with standard guidelines recommended by the Science Council of Japan. All procedures were performed using standard aseptic techniques, and animals were carefully monitored during recovery from anesthesia. We used male Japanese white rabbits that weighed 2.5–3.0 kg at the time of the experiment.

To test the effect of DHG, intrapericardial adhesion was induced by surgery. The animals induced for intrapericardial adhesion were divided into two groups: without DHG treatment (Adh group, *n* = 19) and with DHG treatment (Adh + DHG group, *n* = 19). Sham operation was performed on 9 animals with the same procedure except the opening of the pericardium and the induction of intrapericardial adhesion. A group of 5 animals without any surgery (Pre-group) was used to observe the tissue without any intervention.

For the induction of intrapericardial adhesion, the animals were anesthetized with 10 mg/kg ketamine and 3 mg/kg xylazine intravenously under spontaneous breathing, and placed in a dorsal position. After removing the chest hair, followed by a midline incision and a sternotomy, taking care not to injure the pericardium and pleura, a pericardiotomy was performed. Then, the anterior and lateral epicardium, and pericardium were scrubbed 10 times with gauze, and the pericardial cavity was exposed to 1 ml of autologous blood with 60 IU of heparin to induce intrapericardial adhesion. Five minutes later, the blood was removed with gauze.

**Fig. 1:**
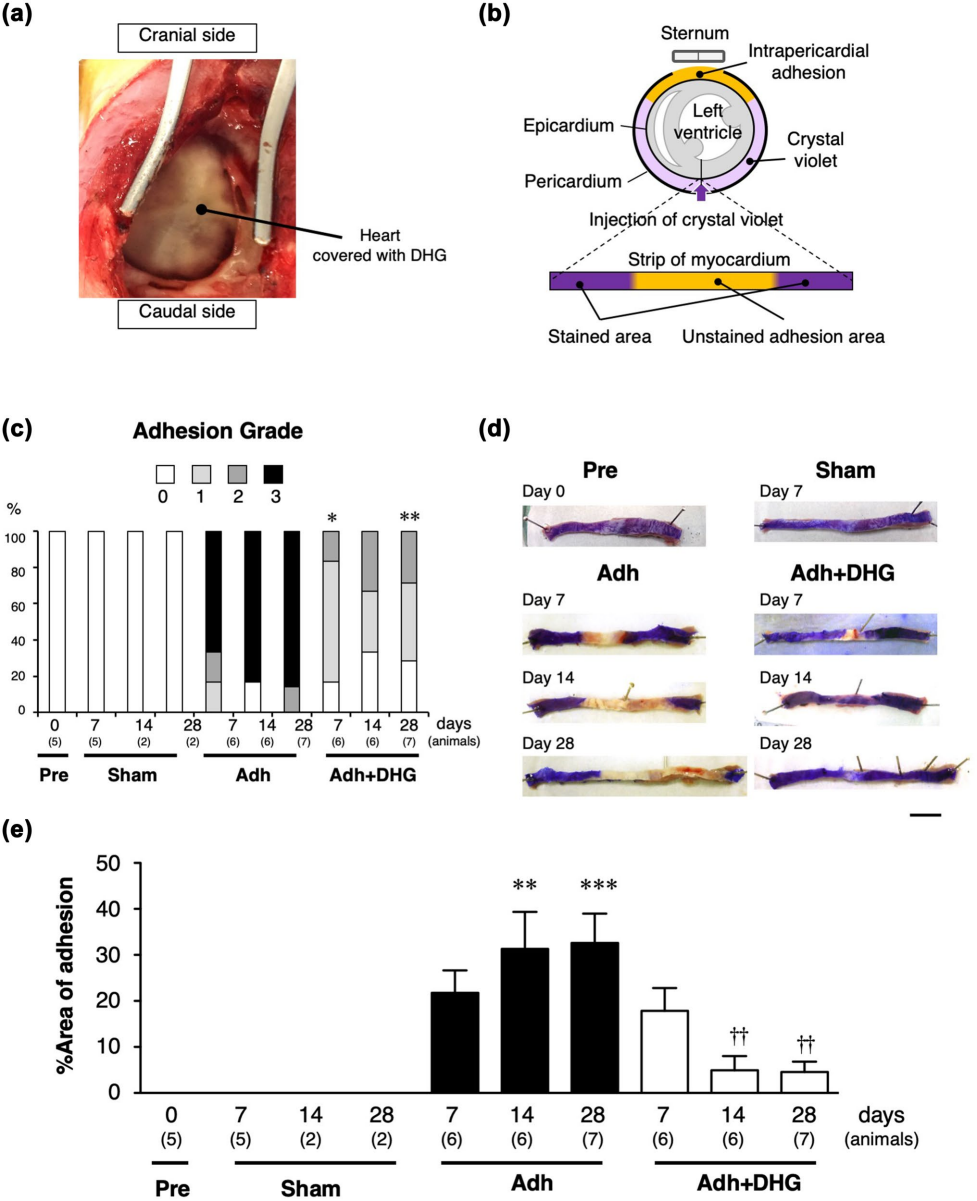
Quantitative assessment of intrapericardial adhesion. **a** Operative view after DHG spray to the anterior wall of the heart through median sternotomy. **b** Schematic diagram for the determination of intrapericardial adhesion area by crystal violet staining. **c** Intrapericardial adhesion score. Grade 0: no adhesion; Grade 1: light and foamy adhesion, Grade 2: intermediate adhesion that could be separated by digital manipulation; Grade 3: dense adhesion that required sharp dissection to separate the pericardium and myocardium. Animals with each grade are shown by percentage in a given experimental group. *p < 0.05, **p < 0.01 compared to the corresponding Adh group by the Fisher’s exact test. **d** Circumferential strips of the ventricular wall with crystal violet staining of the epicardial side on day 0 (Pre; before the induction of adhesion), day 7, day 14, and day 28 after the induction of intrapericardial adhesion. Animals were either untreated (Adh) or treated with DHG (Adh + DHG) after the induction of intrapericardial adhesion. The image of a sham-operated animal, where the pericardium was opened without the induction of adhesion, is also shown. Scale bar: 10 mm. **e** %Area of adhesion was assessed for sham-operated animals and for animals with adhesion induction by the ratio of the unstained area in the total area of the strips of the ventricular wall. Data are shown as mean ± standard error. The number of animals is shown in parentheses. Multiple comparisons were made for all pairs, and for simplicity, the results are indicated only for those with a significant difference. **p < 0.01, ***p < 0.001 compared to sham day 7, ^††^*p* < 0.01 compared to the Adh group at the corresponding time point

For the DHG treatment, solutions A and B of AdSpray (TERUMO, Tokyo, Japan) were sprayed on the epicardial surface of the anterior wall of the heart and the pericardial surface at a pressure of 0.1 MPa according to the manufacturer’s instructions for clinical use in abdominal surgery (Fig. 1a).

After the procedure to induce intrapericardial adhesion and DHG treatment, the pericardium was left open, the sternum was sutured, and the skin was closed. The animals were euthanized without any intervention (Pre), or at the postoperative day 7, 14 and 28 by carbon dioxide inhalation as recommended in the Guidelines for Proper Conduct of Animal Experiments by the Science Council of Japan, and the heart was obtained en bloc with surrounding tissue including the sternum and pericardial sac.

### Evaluation of adhesions

The intrapericardial adhesion was assessed according to the scoring system previously reported [18] as follows: Grade 0: no adhesion; Grade 1: light and foamy adhesion, Grade 2: intermediate adhesion that could be separated by digital manipulation; Grade 3: dense adhesion that required sharp dissection to separate the pericardium and myocardium.

We also developed a method for the evaluation of intrapericardial adhesion using crystal violet, a water-soluble dye. At the time of sacrifice, approximately 0.4 mL aqueous solution of 0.4% crystal violet was gently injected into the posterior surface of the pericardial sac without any adhesion until the pericardial cavity was slightly distended (Fig. 1b), and left for 1 min. Then, the crystal violet solution was washed out from the injection site. Crystal violet stained the free surface of the pericardial cavity with no adhesion, leaving the surface with intrapericardial adhesion unstained. The ventricles were cut at the level of the papillary muscle for the transverse slices with 5 mm thickness. The free wall of the left ventricle was cut at the center of the posterior wall to make a circumferential strip of the ventricular wall. The intrapericardial adhesion area was quantitated by calculating the ratio of the unstained adherent area to the total area of the ventricular wall strips. A preliminary study showed that the adherent area was smaller than the area of epicardial inflammation (data not shown), suggesting that only a part of the inflammatory tissue became adherent.

### Bromodeoxyuridine uptake assay

To evaluate the inflammatory and wound repair response during the formation of intrapericardial adhesion, we assessed cell proliferation based on the uptake of bromodeoxyuridine (BrdU), a thymidine analog. The BrdU solution (250 mg/mL, Sigma-Aldrich, St Louis, Missouri, USA) was prepared in sterile 0.5 mol/L NaOH/NaHCO_3_ buffer (pH 9.8), and continuously infused using an osmotic pump with a 14-day infusion (2ML-2; DURECT, Cupertino, California, USA) that was implanted into the abdominal cavity. The pumps were implanted either at the time of chest surgery or 14 days after the surgery to examine the timing of cell proliferation after the induction of intrapericardial adhesion. The animals with osmotic pump implantation at the time of surgery were sacrificed on day 7 (*n* = 6 in each group), on day 14 (*n* = 6 in each group), and on day 28 (*n* = 4 in each group, termed as “Day 28-first”). The animals with osmotic pump implantation on day 14 were sacrificed on day 28 (*n* = 3 in each group, termed as “Day 28-second”). We also assessed BrdU uptake in the sham group on days 7, 14, and 28. Animals without any intervention or BrdU infusion (Pre-group) served as a negative control.

### Histological observations

After evaluation of the intrapericardial adhesion, a 5-mm slice of the whole heart with pericardium at the level of papillary muscles was fixed in 10% neutralized buffered formalin solution. The samples were embedded in paraffin blocks and sectioned (5 μm). Histochemical staining was performed with hematoxylin–eosin (HE) or Masson trichrome (MT) staining. Immunohistochemical staining was performed using anti-BrdU antibody (Agilent #M0744, Santa Clara, California, USA) and nuclear counterstaining with hematoxylin. For the assessment of the tissue response, the anterior ventricular wall including the pericardium beneath the sternum was divided into three regions: the outer, middle, and inner regions. Digital microscopy images were obtained using a BZ-9000 microscope (Keyence, Osaka, Japan) equipped with a 40X objective lens. The BrdU-positive cell numbers were the average of three digital images with 1360 × 1024 pixels corresponding to 362 × 273 μm from each site, and expressed as the cell numbers in 0.1 mm^2^.

### Statistical analysis

Data analysis was performed using GraphPad PRISM 5 (GraphPad Software, San Diego, California, USA). For scoring adhesion, a statistical analysis was performed using Fisher’s exact test. The results are expressed as means ± standard errors. For other assays, comparisons were made using the Kruskal–Wallis test followed by Dunn’s multiple-comparison test. Significance was set at *p* < 0.05.

## Results

### Macroscopic evaluation of intrapericardial adhesion

DHG was evident on the epicardial and pericardial surface, just after the surgical procedure to induce intrapericardial adhesion and the application of DHG (Fig. 1a), but was invisible on day 7 or later after the procedure. The adhesion score, as determined by the requirement of sharp dissection to isolate the heart [18], revealed that most of the animals in the Adh group showed Grade 3 adhesions, whereas the animals in the Adh + DHG group showed Grades 0–2 and no animal showed Grade 3 adhesions (Fig. 1c). The grading score of the Adh + DHG group was significantly lower at day 7 and day 28 compared with that of the Adh group. The sham-operated animals showed no intrapericardial adhesion on days 7, 14, or 28. The adhesion area in the Adh group, as determined by the absence of crystal violet staining, was 21.8 ± 4.8%, 31.3 ± 8.0%, and 32.6 ± 6.4% on days 7, 14, and 28, respectively (Fig. 1d–e). Animals in the Adh + DHG group showed 17.9 ± 4.9% adhesion on day 7, which was comparable to that of the Adh group (Fig. 1e). However, the DHG group showed 5.0 ± 2.2% and 4.6 ± 2.2% adhesion on day 14 and day 28, respectively, with these values being significantly lower than the corresponding values in the Adh group. These findings indicated that DHG effectively prevented intrapericardial adhesion.

### Microscopic evaluation of intrapericardial adhesion

**Fig. 2:**
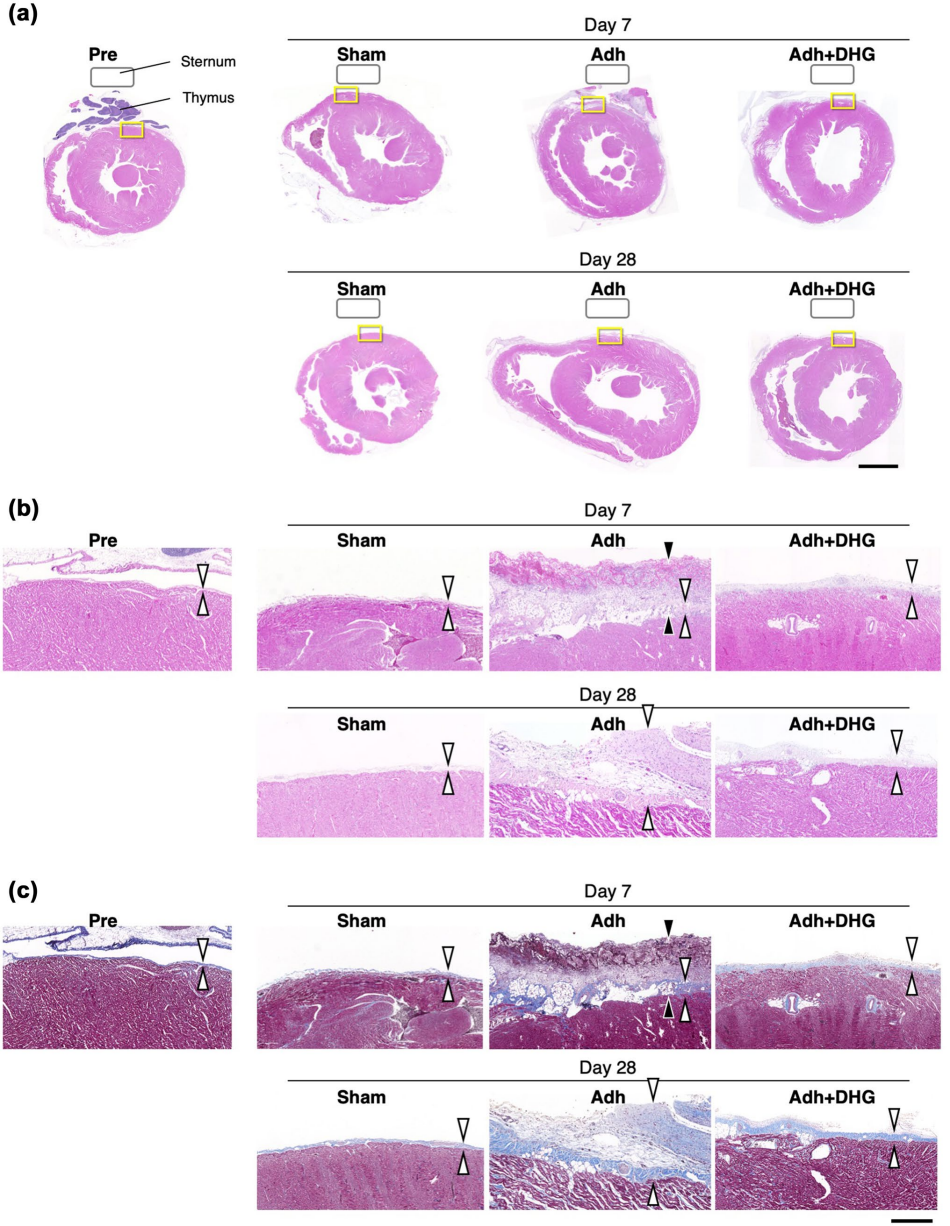
Histopathological findings of intrapericardial adhesion model. Cross-sectional view of the HE-stained hearts on day 7 and day 28 after induction of intrapericardial adhesion alone (Adh), induction of intrapericardial adhesion and DHG treatment (Adh + DHG), or without induction of intrapericardial adhesion (Sham). The sample without any procedure (Pre) is also shown. Scale bar: 5 mm. Rectangles indicate the location of the sternum. **a** Low-magnification images showing the entire cross section. Scale bar: 1 mm. **b** Magnified images with HE staining corresponding to the yellow rectangles in (**a)**. **c** Magnified images with MT staining corresponding to (**b)**. White arrowheads indicate the collagen layers. Black arrowheads indicate adhesive tissue with inflammatory cell infiltration. Scale bar: 200 μm

The hearts of the sham-operated animals without the induction of intrapericardial adhesion showed a smooth outer myocardium. This was covered with a pericardium that contained little cellular components as shown by HE staining (Fig. 2a, b) and was composed of a thin layer of collagenous tissue as shown by MT staining (Fig. 2c). The epicardial tissue in the Adh group, after the induction of intrapericardial adhesion, showed strong cellular infiltration and a mesh of collagenous fibers on day 7. The tissue in the Adh group on day 28 showed a reduction in cellular infiltration, instead, a thick structure of collagenous fibers was observed. In the Adh + DHG group, after the induction of intrapericardial adhesion and DHG treatment, the epicardial tissue showed less cellular infiltration compared with the Adh group on both day 7 and day 28. In addition, the epicardial fibrous tissue was thinner in the Adh + DHG group than in the Adh group, suggesting that there was less inflammation in the Adh + DHG group.

### Evaluation of tissue injury response

**Fig. 3:**
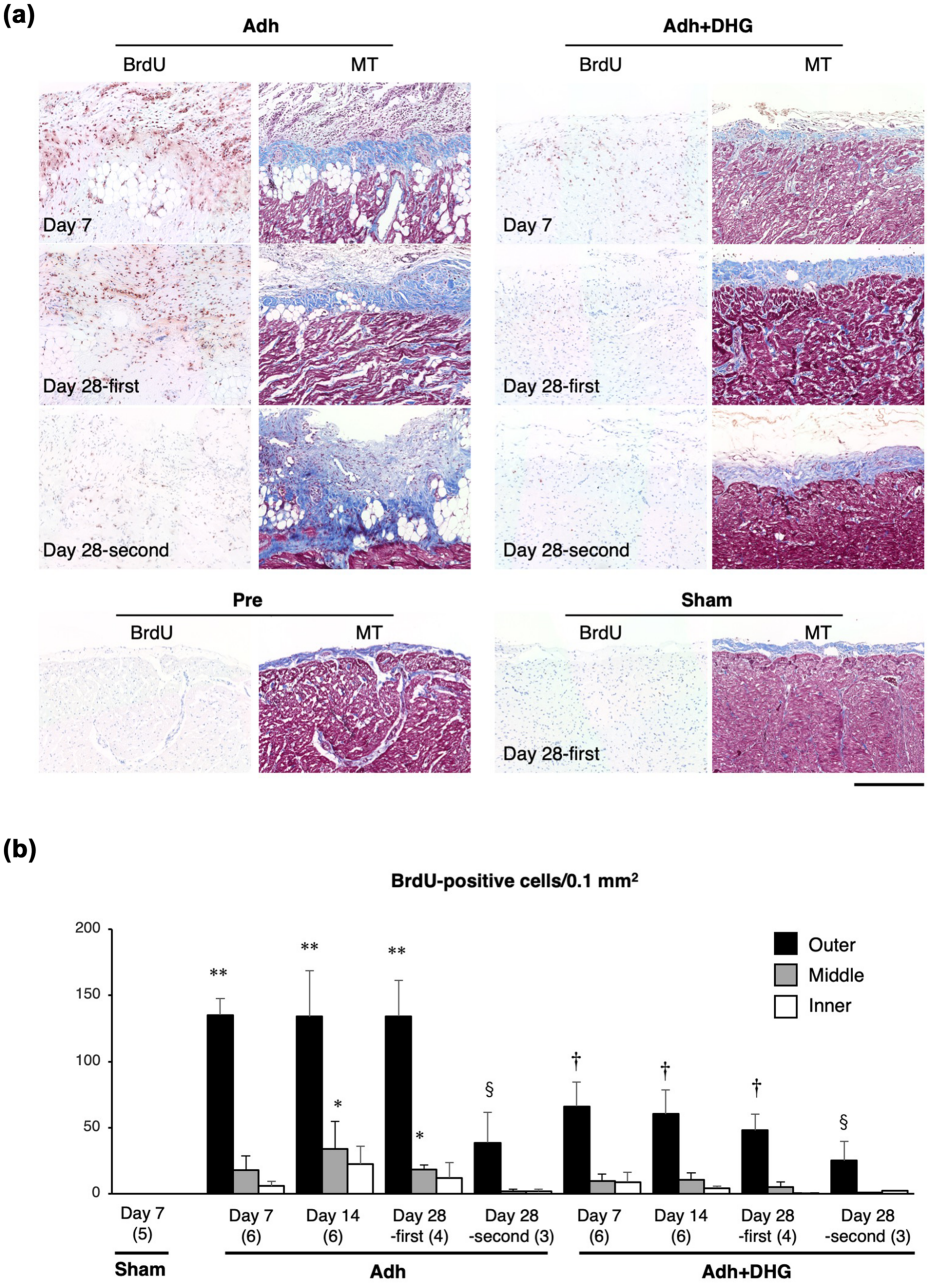
Proliferative response in the outer ventricular wall. **a** Immunohistochemical staining of the outer ventricular wall for BrdU uptake (brown) with hematoxylin counterstaining for nuclei. MT-stained images are shown for the serial sections corresponding to BrdU staining. All of the images are oriented such that the epicardial surface is on top. Samples were obtained from animals before the procedure (Pre), after induction of intrapericardial adhesion alone (Adh), induction of intrapericardial adhesion and DHG treatment (Adh + DHG) on day 7 and day 28. The images for the sham-operated sample (Sham) are also shown. BrdU was administered throughout the time period for day 7 samples. For day 28 samples, BrdU was administered either during the first half (Day 28-first) or the second half (Day 28-s) of the 28-day time period. Scale bar: 200 μm. **b** BrdU-positive cell numbers in 0.1 mm^2^ were determined in the outer, middle, and inner regions of the ventricular walls. For the Day 7 and Day 14 samples, BrdU was administered throughout the time period. For the Day 28 samples, BrdU was administered during the first (Day 28-first) or the second 14 days (Day 28-s). Data are shown as mean ± standard error. The number of animals is shown in parentheses. Multiple comparisons were made for all pairs in the corresponding site and only those with a significant difference are indicated for simplicity. *p < 0.05, **p < 0.01 compared to Sham, ^†^*p* < 0.05 compared to the corresponding time point in Adh, ^§^*p* < 0.05 compared to Adh Day 28-first

BrdU uptake, an indicator of cell proliferative response, was undetectable in the ventricular wall in sham-operated animals (Fig. 3). BrdU-positive cells appeared mainly in the outer region of the ventricular wall, and to a lesser extent in the middle as well as in the inner region of the ventricular wall (Fig. 3b). Therefore, we focused on the response in the outer ventricular wall, including adhesive tissue just below the sternum, where the injury response was most prominent. In the Adh group, the number of BrdU-positive cells was 136.8 ± 11.3, 135.5 ± 31.8, and 135.7 ± 23.8 cells/0.1 mm^2^ on days 7, 14, and 28, respectively, when BrdU administration was started on the day of the induction of intrapericardial adhesion. The number of BrdU-positive cells was reduced to 39.0 ± 19.2 cells/0.1 mm^2^ on day 28 when BrdU was administered only during the last 14 days of the 28-day observation period (Day 28-s in Fig. 3b), indicating that cell proliferation was decreased during this period. In the Adh + DHG group, the number of BrdU-positive cells was decreased to 66.8 ± 17.2, 61.0 ± 16.9, and 48.6 ± 10.7 cells/0.1 mm^2^ on days 7, 14, and 28, respectively, compared to Adh group (Fig. 3b), indicating that the treatment of animals with DHG resulted in a significant reduction of BrdU-positive cells.

## Discussion

In this study, we demonstrated that DHG effectively prevented intrapericardial adhesion in an animal model. DHG-treated animals showed looser intrapericardial adhesion that could be easily dissociated from the heart, as assessed by the adhesion score. The adhesion area, as assessed by crystal violet staining, was also much lower in the DHG-treated animals. Notably, although the adhesion area was comparable between the Adh and Adh + DHG groups on day 7, the adhesion score was lower in the Adh + DHG group. On day 28, both adhesion score and adhesion area were lower in the Adh + DHG group than in the Adh group. Histologically, the Adh + DHG group showed lower cellular infiltration and BrdU-positive cells, indicating that DHG attenuated the injury response.

Our results showed that intrapericardial adhesions are associated with the activation of cell proliferation. Although we did not specifically determine the cell types in the current study, previous studies have shown that pericardial mesodermal cells, fibroblasts, and inflammatory cells proliferate during the early phase of intrapericardial adhesion formation [8–10]. The BrdU uptake assay showed that the number of BrdU-positive cells was comparable among the samples on days 7, 14, and 28 when BrdU administration was started at the time of the induction of intrapericardial adhesion but was reduced when BrdU was administered only during the second half of the 28-day observational period. Therefore, it seems that the proliferative response occurred within 7 days after the induction of intrapericardial adhesion, and diminished thereafter. This time course is consistent with the well-established sequence of events in the wound healing process [19].

In the Adh + DHG group, although the adhesion area was comparable to that of the Adh group on day 7, both the adhesion score and the count of BrdU-positive cells were lower than those in the Adh group. The adhesion area and score were lower in the Adh + DHG group than in the Adh group on day 28. These findings suggest that a loose adhesion was formed on day 7, which prevented crystal violet staining in the Adh + DHG group, and the adhesion was resolved subsequently, possibly because the injury response was suppressed by DHG, as demonstrated by the lower number of BrdU-positive cells on day 7. While DHG forms a thick coating over the sprayed surface, it is disassembled and absorbed within 3 days in the abdominal cavity [12, 14]. Accordingly, DHG was not observed in the pericardial cavity by visual inspection 7 days after the initial DHG spray, although formal proof of DHG degradation in the pericardial cavity requires further study. Summarily, we observed that placing the DHG barrier for a short period after the surgical procedure was sufficient to prevent the injury response and firm intrapericardial adhesion in our experimental setting.

Several issues need to be addressed before the clinical use of DHG for preventing intrapericardial adhesion. First, the safety of DHG use needs to be tested in the context of cardiac surgery. Although the general safety of DHG is established in surgeries performed in the abdominal cavity, the biological response may be different in the pericardial cavity. As the physical capacity of the pericardial cavity is significantly smaller than that of the abdominal cavity, and due to the risk of causing cardiac tamponade [8], the volume of DHG would need to be carefully adjusted. Second, the fate of DHG in the pericardial cavity needs to be investigated under various conditions such that it lasts sufficiently long to prevent intrapericardial adhesion, but is degraded and absorbed early enough to avoid unexpected adverse effects. Third, the efficacy of DHG in preventing the adhesion of artificial materials such as vascular grafts and patches needs to be evaluated. Fourth, a non-invasive method to evaluate intrapericardial adhesion, possibly by an imaging technique, needs to be established to test if DHG is effective before planning clinical trials.

## Conclusion

Although further research is required before introducing DHG to clinical practice, our results demonstrated that it effectively prevented postoperative intrapericardial adhesion in the animal model. Prevention of intrapericardial adhesion would benefit patients who undergo repeated cardiac surgeries.
